# Restricted Cell Surface Expression of Receptor Tyrosine Kinase ROR1 in Pediatric B-Lineage Acute Lymphoblastic Leukemia Suggests Targetability with Therapeutic Monoclonal Antibodies

**DOI:** 10.1371/journal.pone.0052655

**Published:** 2012-12-20

**Authors:** Hema Dave, Miriam R. Anver, Donna O. Butcher, Patrick Brown, Javed Khan, Alan S. Wayne, Sivasubramanian Baskar, Christoph Rader

**Affiliations:** 1 Experimental Transplantation and Immunology Branch, Center for Cancer Research, National Cancer Institute, National Institutes of Health, Bethesda, Maryland, United States of America; 2 Pediatric Oncology Branch, Center for Cancer Research, National Cancer Institute, National Institutes of Health, Bethesda, Maryland, United States of America; 3 Pathology/Histotechnology Laboratory, Science Applications International Corporation–Frederick, Frederick National Laboratory for Cancer Research, National Cancer Institute, National Institutes of Health, Frederick, Maryland, United States of America; 4 Department of Oncology and Pediatrics, Sidney Kimmel Comprehensive Cancer Center, Johns Hopkins Hospital, Baltimore, Maryland, United States of America; 5 Department of Cancer Biology and Department of Molecular Therapeutics, The Scripps Research Institute, Jupiter, Florida, United States of America; Institut national de la santé et de la recherche médicale (INSERM), France

## Abstract

**Background:**

Despite high cure rates for pediatric B-lineage acute lymphoblastic leukemia (B-ALL), short-term and long-term toxicities and chemoresistance are shortcomings of standard chemotherapy. Immunotherapy and chemoimmunotherapy based on monoclonal antibodies (mAbs) that target cell surface antigens with restricted expression in pediatric B-ALL may offer the potential to reduce toxicities and prevent or overcome chemoresistance. The receptor tyrosine kinase ROR1 has emerged as a candidate for mAb targeting in select B-cell malignancies.

**Methodology and Principal Findings:**

Using flow cytometry, Western blotting, immunohistochemistry, and confocal immunofluorescence microscopy, we analyzed the cell surface expression of ROR1 across major pediatric ALL subtypes represented by 14 cell lines and 56 primary blasts at diagnosis or relapse as well as in normal adult and pediatric tissues. Cell surface ROR1 expression was found in 45% of pediatric ALL patients, all of which were B-ALL, and was not limited to any particular genotype. All cell lines and primary blasts with E2A-PBX1 translocation and a portion of patients with other high risk genotypes, such as MLL rearrangement, expressed cell surface ROR1. Importantly, cell surface ROR1 expression was found in many of the pediatric B-ALL patients with multiply relapsed and refractory disease and normal karyotype or low risk cytogenetics, such as hyperdiploidy. Notably, cell surface ROR1 was virtually absent in normal adult and pediatric tissues.

**Conclusions and Significance:**

Collectively, this study suggests that ROR1 merits preclinical and clinical investigations as a novel target for mAb-based therapies in pediatric B-ALL. We propose cell surface expression of ROR1 detected by flow cytometry as primary inclusion criterion for pediatric B-ALL patients in future clinical trials of ROR1-targeted therapies.

## Introduction

Pediatric B-ALL is the most common childhood cancer in the USA, accounting for ∼25% of all cancers. Pediatric B-ALL generally arises from pre-B cells in bone marrow and has the general immunophenotype CD10+ CD19+, yet its genotypes differ widely [1]. For example, one third of cases have chromosomal translocations, including t(12;21), t(1;19), t(9;22), and t(4;11), which generate the fusion oncogenes TEL-AML1, E2A-PBX1, BCR-ABL, and MLL-AF4, respectively. Other common cases of pediatric B-ALL have hyperdiploid, hypodiploid, and complex genotypes. Cure rates for pediatric B-ALL are >80% with optimal use of chemotherapy based on risk-based stratification [2]. However, the survival for the 15–20% of children who relapse is short and survivors have significant risks of long-term toxicities from chemotherapy, including secondary cancers, cardiovascular disease, obesity, neurocognitive and psychosocial disorders, and sterility.

Therapies that selectively target malignant B cells in pediatric B-ALL have the potential to reduce short-term and long-term toxicities, and to overcome chemotherapy resistance. Several B-lineage cell surface differentiation antigens expressed by B-ALL blasts have been targeted with monoclonal antibody (mAb)-based therapies in clinical trials and demonstrate proof-of-principle of the potential for efficacy [3]. For example, CD22 is targeted by naked mAb epratuzumab [4], antibody-drug conjugate inotuzumab ozogamicin [5], [6] and immunotoxin moxetumomab pasudotox [7], and CD19 is targeted by bispecific T-cell engaging antibody blinatumomab [8], [9]. However, the expression of CD19, CD22, and all other currently targeted cell surface antigens is not restricted to B-ALL blasts, but shared with normal B cells.

Gene expression profiling identified ROR1, a receptor tyrosine kinase predominantly expressed in embryogenesis [10], as a signature gene in chronic lymphocytic leukemia (CLL) [11], [12], which we and others confirmed by a comprehensive analysis of ROR1 protein expression [13]–[15]. We also showed that ROR2, which shares 58% amino acid sequence identity with ROR1 and the only other member of the ROR family [10], is not expressed by primary CLL cells [13]. Subsequently, it was found that ROR1 is also expressed in certain other B-cell malignancies, such as mantle cell lymphoma and marginal zone lymphoma [16], [17]. Importantly, normal B cells, other normal circulating cells, and normal adult tissues, with few exceptions [17], [18], did not reveal expression of cell surface ROR1.

An interesting exception is an intermediate stage of normal bone marrow CD10+ CD19+ CD34-negative TdT-negative pre-B cells, which express ROR1 at similar levels as primary CLL cells [18]. This recent finding, along with reports of ROR1 mRNA expression in primary B-ALL blasts [19], prompted an investigation of cell surface ROR1 expression in B-ALL. Interestingly, a subtype of B-ALL defined by a t(1;19) chromosomal translocation that generates the oncogenic fusion protein E2A-PBX1, revealed uniform (4/4) expression of cell surface ROR1, whereas only a small fraction (2/35) of t(1;19)-negative cases were positive [18]. Evidence suggesting a functional role of ROR1 in B-ALL came from an siRNA study that systematically knocked down all tyrosine kinases in a panel of primary leukemia cells; in a t(1;19) B-ALL case, ROR1 emerged as the only tyrosine kinase that, when targeted with siRNA, significantly decreased the *ex vivo* viability of primary B-ALL blasts [20].

To establish a rationale and platform for targeting ROR1 with mAb-based therapies in B-ALL, the current study employed flow cytometry, Western blotting, immunohistochemistry (IHC), and confocal immunofluorescence microscopy. Cell surface expression of ROR1 was analyzed across major pediatric B-ALL subtypes represented by 14 cell lines and 56 primary blasts as well as in normal adult and pediatric tissues.

## Results

### ROR1 mRNA and Protein is Expressed in Pediatric ALL

**Figure 1:**
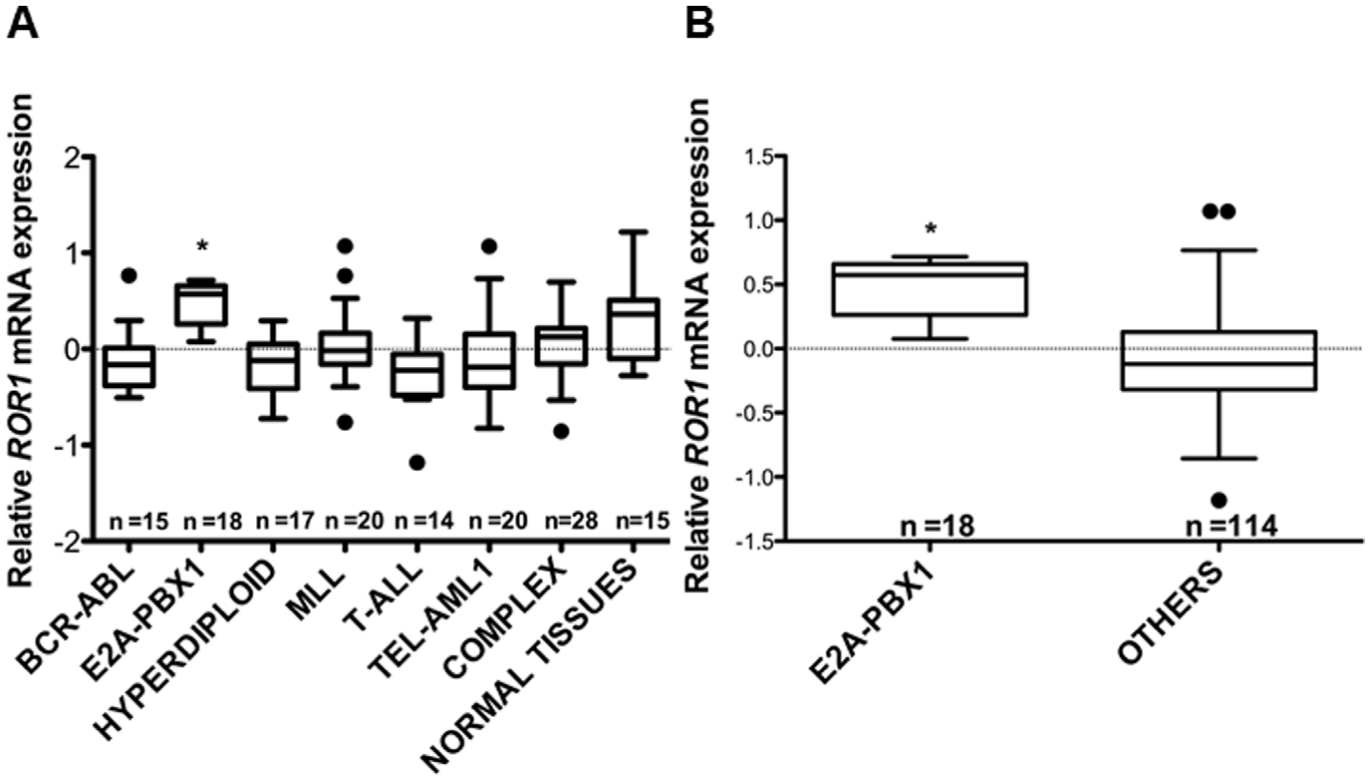
ROR1 mRNA expression in pediatric ALL. (**A**) Log_2_ median-centered gene expression profiling data for ROR1 mRNA isoform 1 from a previously published dataset representing 132 pediatric patients with newly diagnosed ALL (www.stjuderesearch.org/data/ALL1) [21] revealed uniform expression in the E2A-PBX1 group (p<0.0001, one-way ANOVA test) and variable expression in all other groups. ROR1 mRNA isoform 1 expression was also detected in normal tissues. (**B**) ROR1 mRNA isoform 1 expression was significantly higher in the E2A-PBX1 group compared to all other groups combined (p = 0.0001, Mann Whitney test).

Two splice variants of ROR1 mRNA exist. Isoform 1 encodes the complete ROR1 protein with the three extracellular domains followed by the transmembrane and cytoplasmic segments. Isoform 2 encodes only the extracellular segment. We analyzed ROR1 mRNA isoform 1 expression in a previously published dataset of 132 pediatric patients with newly diagnosed ALL, including B-ALL and T-lineage ALL (T-ALL) (www.stjuderesearch.org/data/ALL1) [21]. Whereas the median ROR1 mRNA isoform 1 expression level was only −0.0406 across all 132 patients, 62 patients (47%) had levels higher than an arbitrary cutoff of zero (Fig. 1A). Notably, ROR1 mRNA isoform 1 was expressed in all 18 patients with E2A-PBX1 genotype. ROR1 mRNA expression in the E2A-PBX1 group was significantly higher (median  = 0.5733) than in all other ALL groups combined (median = −0.1199; p<0.0001) (Fig. 1B). Nonetheless, among the other groups, BCR-ABL, MLL, TEL-AML1, and complex genotype each included at least one case with ROR1 mRNA isoform 1 expression levels at or above the median of the E2A-PBX1 group (Fig. 1A). Kaplan-Meier survival curves for ROR1+ and ROR1-negative pediatric B-ALL patients revealed no difference regardless of whether (p = 0.334) or whether not (p = 0.452) the E2A-PBX1 group was included. Thus, similar to CLL [13] and contrary to breast cancer [22], ROR1 mRNA expression in pediatric B-ALL is not associated with aggressive disease. The dataset also included 15 normal tissues, which combined revealed a higher median ROR1 mRNA isoform 1 expression level than all ALL groups with the exception of E2A-PBX1 (Fig. 1A).

**Figure 2:**
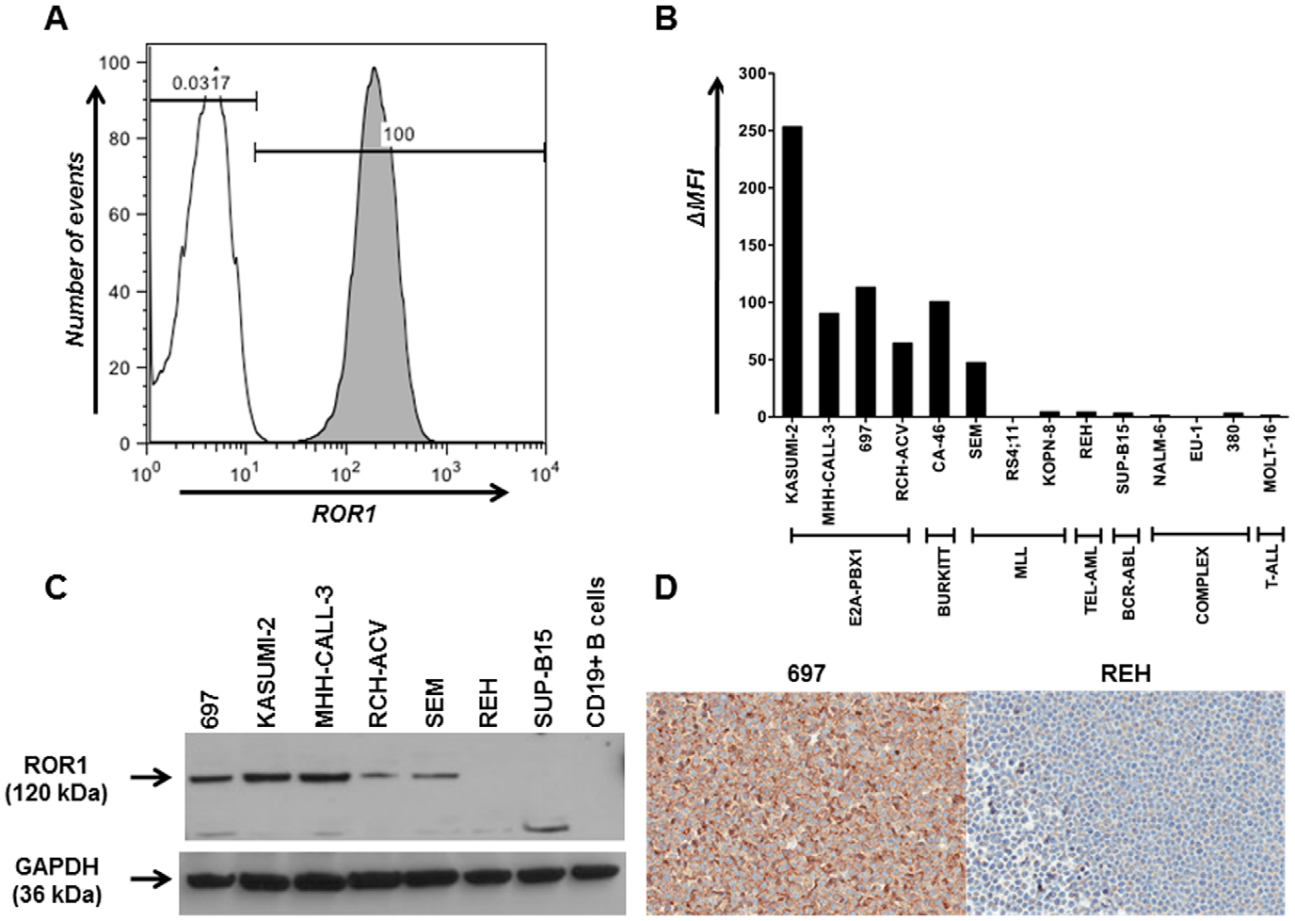
Cell surface ROR1 expression in ALL cell lines. (**A**) Flow cytometry profile of pre-B-ALL cell line 697 stained with mouse anti-human ROR1 mAb 2A2 (gray) or mouse IgG1 as isotype control (white) followed by DyLight 649-conjugated goat anti-mouse IgG pAbs. (**B**) Using the same method, 14 ALL cell lines that represent the immunophenotypic and genotypic heterogeneity in ALL were analyzed for cell surface ROR1 expression. The ΔMFI was calculated as the difference of MFI values obtained for mAb 2A2 and isotype control. Six ALL cell lines revealed cell surface ROR1 expression. (**C**) Protein lysates (10 µg/lane) from the 5 positive pre-B-ALL cell lines, but not from negative cell lines and normal B cells, revealed a ∼120 kDa band by Western blotting using polyclonal goat anti-human ROR1 pAbs followed by HRP-conjugated donkey anti-goat IgG pAbs. The membrane was stripped and probed with rabbit anti-human GAPDH (∼36 kDa) pAbs followed by HRP-conjugated goat anti-rabbit IgG pAbs to confirm uniform loading of protein lysates. (**D**) Immunohistochemical analysis of FFPE slides of pre-B-ALL cell lines 697 (left) and REH (right) probed with goat anti-human ROR1 pAbs. (Magnification 20×).

To investigate whether these findings correlated with ROR1 expression on the cell surface, we first analyzed 14 cell lines representing the immunophenotypic and genotypic heterogeneity in ALL. Flow cytometry using mouse anti-human ROR1 mAb 2A2 (Fig. 2A and B) or goat anti-human ROR1 pAbs (data not shown) revealed cell surface ROR1 expression in 5 pre-B-ALL cell lines, including all 4 E2A-PBX1+ cell lines and 1 out of 3 MLL-rearranged cell lines. Representing mature-B-ALL or Burkitt ALL, the Burkitt lymphoma cell line CA-46 was also positive. Protein lysates from cell lines with cell surface ROR1 expression revealed a 120-kDa band by Western blotting using goat anti-human ROR1 pAbs, which was not detectable in negative cell lines and normal B cells (Fig. 2C). Cell surface ROR1 expression was further confirmed by IHC using goat anti-human ROR1 pAbs on a formalin-fixed paraffin-embedded (FFPE) of pre-B-ALL cell line 697 with E2A-PBX1 genotype, whereas neither cell surface nor intracellular ROR1 expression was detectable in the cell line REH with TEL-AML1 genotype (Fig. 2D).

**Figure 3:**
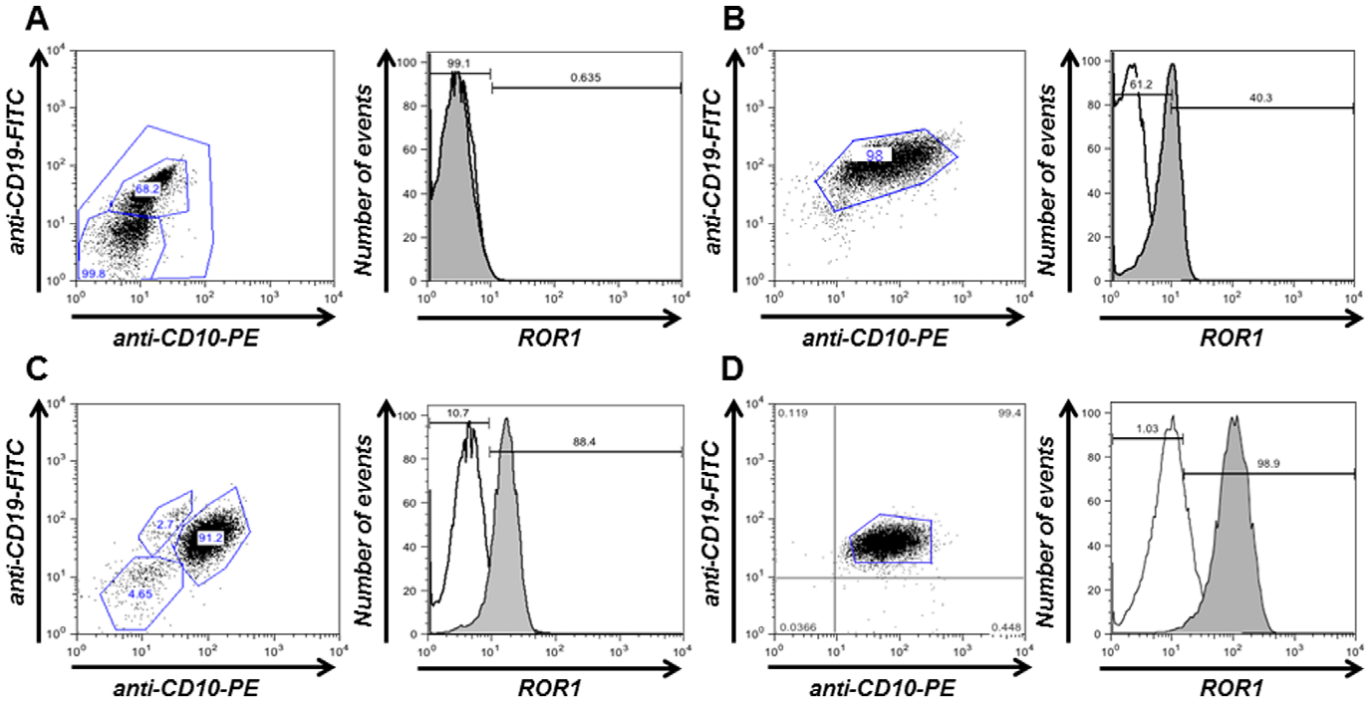
Cell surface ROR1 expression on primary ALL blasts. (**A**) Flow cytometry profiles of primary ALL blasts from B-ALL patients showing the various intensities of cell surface ROR1 expression. (**A**) Representative ROR1-negative sample. (**B**), (**C**), and (**D**) ROR1+ samples with MFI scores “+”, “++”, and “+++”, respectively. (MFI score: 0, ΔMFI <2; +, ΔMFI >2 and <5; ++, ΔMFI >5 and <10; +++, ΔMFI >10). Four-color flow cytometry was done by first staining with mouse anti-human ROR1 mAb 2A2 (gray) or mouse IgG (white) as isotype control followed by DyLight 649-conjugated goat anti-mouse IgG pAbs, addition of propidium iodide to exclude dead cells from the analysis, and gating with an FITC-conjugated anti-human CD19 mAb and a PE-conjugated anti-human CD10 mAb. Primary ALL blasts in the CD19+ CD10+ gate (left) uniformly expressed cell surface ROR1 (right).

**Figure 4:**
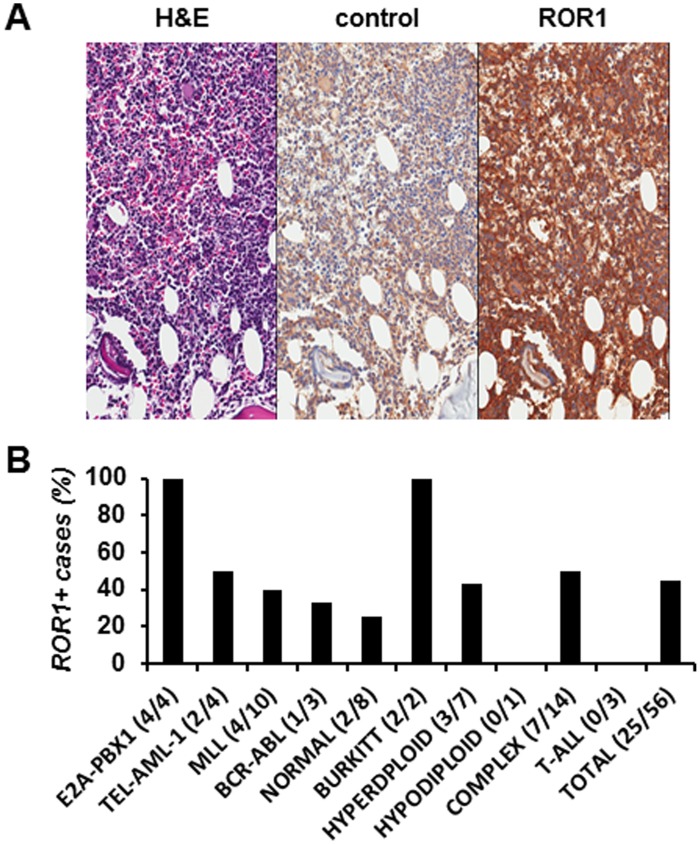
Cell surface ROR1 expression on primary ALL blasts. (**A**) Immunohistochemical analysis of FFPE slides of bone marrow from a pre-B-ALL patient with hyperdiploid genotype (ID 35; Table 1) probed with normal goat pAbs (control; middle panel) and goat anti-human ROR1 pAbs (ROR1; right panel). The nuclei of ALL blasts were identified by hematoxylin and eosin staining (H&E; left panel). (**B**) Proportion of ROR1+ cases by genotype based on 56 pediatric ALL patients (Table 1).

We next sought to detect cell surface ROR1 in primary ALL blasts from 56 pediatric patients with heterogeneous immunophenotypes and genotypes by flow cytometry. Primary ALL blasts were gated using lineage specific markers CD19 and CD10 (B-ALL) and CD3 and CD5 (T-ALL). Using mAb 2A2, 17 out of 44 samples (39%) revealed ΔMFI (mean fluorescence intensity) values that exceeded the isotype control by at least 2-fold (Table 1) (Fig. 3). The patients were scored based on ΔMFI values as shown in Table 1 and Fig. 3. Six patients had a score of “+” (ΔMFI >2 and <5), 7 patients a score of “++” (ΔMFI >5 and <10), and 4 patients a score of “+++” (ΔMFI >10). Bone marrow IHC in 8 out of 12 patients detected cell surface ROR1 in primary ALL blasts (Fig. 4A and Table 1). Quantitative flow cytometry based on PE-conjugated mAb 2A2 and QuantiBRITE PE beads as standard, estimated the number of ROR1 molecules on the cell surface of primary ALL blasts from three patients with MFI scores of “+”, “++”, and “+++” at 2,494, 3,715, and 4,591, respectively (Table 1). Thus, the cell surface density of ROR1 on primary ALL blasts and primary CLL cells are comparable [13]. Collectively, 25 out of 56 patients (45%) revealed cell surface expression of ROR1 (Table 1 and Fig. 4B). Among the 25 positive samples were 4 out of 4 patients with E2A-PBX1, 2 out of 4 with TEL-AML1, 4 out of 10 with MLL, 1 out of 3 with BCR-ABL, 2 out of 8 with normal, 3 out of 7 with hyperdiploid, and 7 out of 14 with complex genotype. One positive sample, which tested negative by multiplex RT-PCR for TEL-AML1, BCR-ABL, E2A-PBX1, and MLL [23], was considered to have complex genotype. The remaining 2 positive samples were from patients with Burkitt ALL. Among the 31 negative samples were 1 out of 1 patient with hypodiploid genotype and 3 out of 3 with T-ALL (Table 1 and Fig. 4B). Collectively, cell surface ROR1 expression correlated well with ROR1 mRNA isoform 1 expression, with consistent expression in the E2A-PBX1 genotype and variable expression in all other subtypes. Notably, the majority of positive samples including 3 out of 4 with a score of “+++” came from pediatric patients with a genotype other than E2A-PBX1.

**Table 1:** Origin and characteristics of primary ALL blasts.

Patient ID	Age (years)	Immunophenotype	Genotype	Status^1^	ΔMFI	MFI score^2^	ABC^3^
**ROR1+**							
1	11	Pre-B	E2A-PBX1	D	11.42	+++	
2	2	Pre-B	E2A-PBX1	D	2.28	+	2494
3	11	Pre-B	E2A-PBX1	R	IHC+^4^		
4	0.8	Pre-B	E2A-PBX1	D	6.87	++	
5	9	Pre-B	TEL-AML1	R	9.2	++	
6	3	Pre-B	TEL-AML1	D	3.7	+	
9	21	Mixed lineage	MLL	R	18.77	+++	4591
10	0.7	Mixed lineage	MLL, infant	R	6.62	++	
16	12	Mixed lineage	MLL	R	IHC+		
17	0.19	Mixed lineage	MLL, infant	R	IHC+		
21	Unknown	Pre-B	BCR-ABL	D	4.32	+	
22	16	Pre-B	Normal	R	IHC+		
28	12	Pre-B	Normal	R	IHC+		
30	17	Mature-B	Burkitt	R	52.54	+++	
31	13	Mature-B	Burkitt	D	8.6	++	3715
35	17	Pre-B	Hyperdiploid	R	IHC+		
36	21	Pre-B	Hyperdiploid	D	2.16	+	
38	15	Pre-B	Hyperdiploid	D	5.16	++	
41	7	Pre-B	Complex	R	4.1	+	
42	19	Pre-B	Complex	R	90.67	+++	
45	9	Pre-B	Complex	R	3.06	+	
46	10	Pre-B	Complex	R	9.38	++	
47	16	Biphenotypic	Complex	R	IHC+		
48	11	Pre-B	Complex^5^	R	IHC+		
51	Unknown	Pre-B	Complex^6^	D	8.33	++	
**ROR1-negative**							
N = 2	2–10	Pre-B	TEL-AML1	D	<2	0	
N = 6	0.1–0.4	Mixed lineage	MLL	D	<2	0	
N = 2	4,5	Pre-B	BCR-ABL	R	<2	0	
N = 6	6–24	Pre-B	Normal	R	<2^7^	0	
N = 4	5	Pre-B	Hyperdiploid	R	<2	0	
N = 1	9	Pre-B	Hypodiploid	R	<2^8^	0	
N = 4	9,12,15,19	Pre-B	Complex	R	<2^9^	0	
N = 3	11–17	Pre-B	Complex^5^	R	<2	0	
N = 3	7–17	T	T-ALL	D	<2	0	

1 Status: D, diagnosis; R, relapse.

2 MFI score: 0, ΔMFI <2; +, ΔMFI >2 and <5; ++, ΔMFI >5 and <10; +++, ΔMFI >10.

3 ABC, antibody bound per cell.

4 Bone marrow tested positive by immunohistochemistry (IHC).

5 AML1 amplified.

6 Tested negative by multiplex RT-PCR for TEL-AML1, BCR-ABL, E2A-PBX1, and MLL [21].

7 Including IHC-negative bone marrow from 2 patients.

8 IHC-negative bone marrow.

9 Including IHC-negative bone marrow from 1 patient.

### Cell Surface ROR1 is Absent in Normal Adult and Pediatric Tissues

To evaluate the targetability of a cell surface antigen for mAb-based therapies, its expression on normal tissues requires close examination. The noted ROR1 mRNA isoform 1 expression across a panel of normal tissues that was detected by gene expression profiling (Fig. 1A) and confirmed by real-time quantitative RT-PCR (data not shown), prompted us to analyze ROR1 protein expression in normal tissues by IHC and Western blotting.

**Figure 5:**
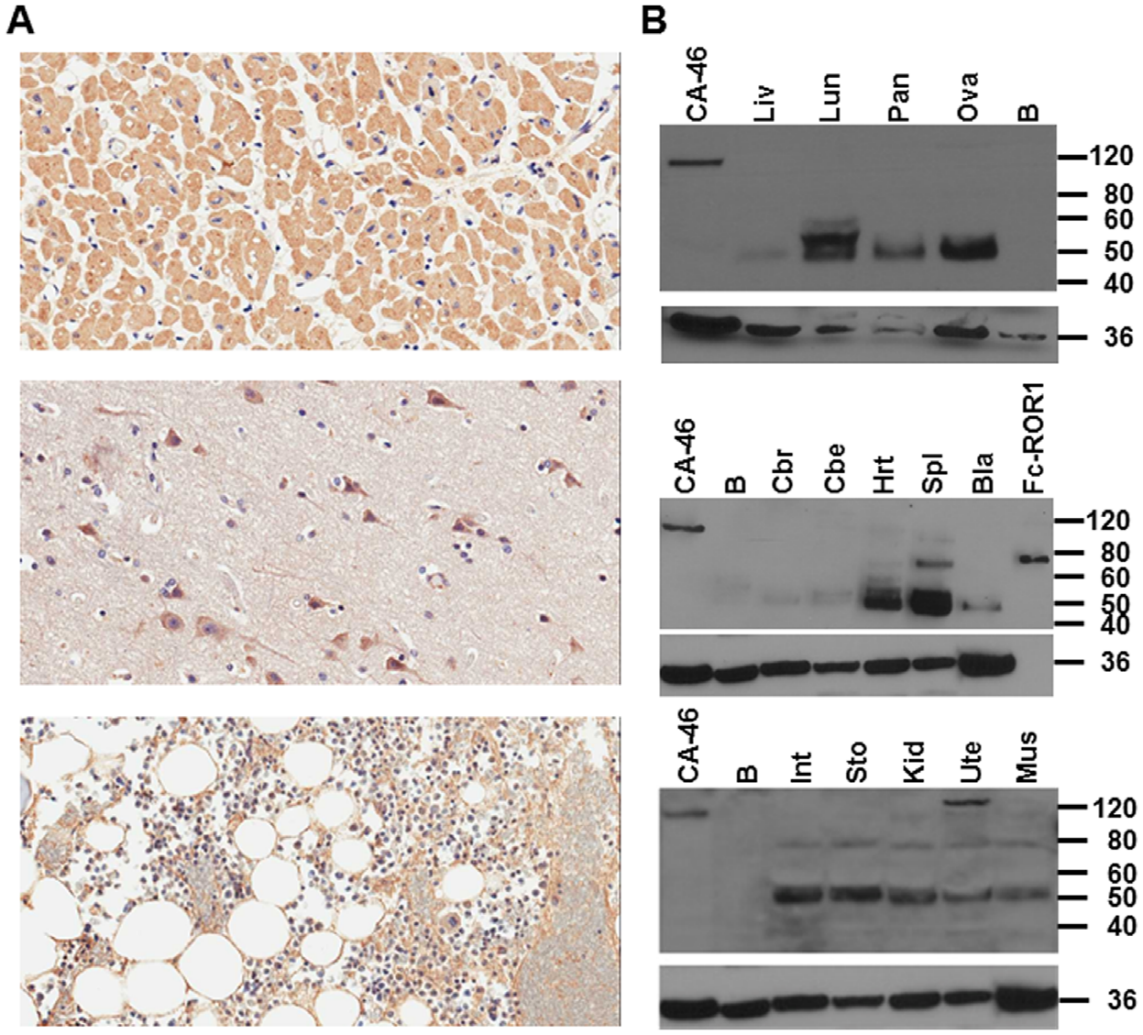
Absence of cell surface ROR1 in normal adult and pediatric tissues. (**A**) Immunohistochemical analysis of FFPE slides of a selection of normal adult tissues (top, heart; middle, cerebrum; bottom, adipocytes) probed with goat anti-human ROR1 pAbs revealed cytoplasmic staining in cardiomyocytes and neurons and plasma membrane staining in adipocytes. (Magnification 20×). (**B**) A protein lysate (5 µg/lane) from Burkitt lymphoma cell line CA-46 was compared to protein lysates (20 µg/lane) from 14 normal pediatric tissues by Western blotting using polyclonal goat anti-human ROR1 pAbs followed by HRP-conjugated donkey anti-goat IgG pAbs. None of the normal pediatric tissues revealed a firm ∼120 kDa band indicative of cell surface ROR1 expression although a faint ∼120 kDa band was detected in heart (Hrt), kidney (Kid), and uterus (Ute). Bands of higher and lower molecular weight are discussed in the text. The previously described [25] recombinant fusion protein Fc-ROR1, which runs as ∼75-kDa band under reducing conditions, was used as additional control. The membrane was stripped and probed with rabbit anti-human GAPDH (∼36 kDa) followed by HRP-conjugated goat anti-rabbit IgG pAbs to confirm uniform loading of protein lysates. Abbreviations: Liv, liver; Lun, lung; Pan, pancreas; Ova, ovary; B, CD19+ B cells; Cbr, cerebrum; Cbe, cerebellum; Hrt, heart; Spl, spleen; Bla, bladder; Int, small intestine; Sto, stomach; Kid, kidney; Ute, uterus; Mus, skeletal muscle.

For IHC, we stained a normal adult tissue array on an FFPE slide with goat anti-human ROR1 pAbs followed by biotinylated rabbit anti-goat IgG pAbs. Whereas ROR1 protein expression was detected in more than half of 32 normal adult tissues, the staining was predominantly cytoplasmic (Table 2). This cytoplasmic staining was typically confined to certain cells within the tissues, such as neurons in the cerebrum (Table 2 and Fig. 5A) and islet cells in the pancreas (Table 2). The only plasma membrane staining was found on adipocytes of the bone marrow (Table 2 and Fig. 5A). It is difficult to distinguish cytoplasmic and plasma membrane staining in adipocytes because they contain a large fat vacuole, which pushes all other organelles and the cytoplasm to the periphery. However, independent evidence for ROR1 protein expression on the cell surface of human and mouse adipocytes was published recently [17], [24].

**Table 2:** ROR1 expression in normal adult tissues as analyzed by immunohistochemistry.

Tissue	Staining pattern
Adrenal gland	cytoplasmic
Bone marrow	membrane (adipocytes)
Breast	none
Cardiac muscle	cytoplasmic
Cerebellum	none
Cerebrum	cytoplasmic (neurons)
Colon	cytoplasmic (enterochromaffin cells)
Endometrium	cytoplasmic (glands)
Esophagus	none
Eye	none
Hypophysis	cytoplasmic
Kidney	cytoplasmic (tubules)
Larynx	cytoplasmic
Liver	cytoplasmic
Lung	cytoplasmic (alveolar macrophages)
Mesothelium	none
Ovary	cytoplasmic (stroma)
Pancreas	cytoplasmic (islet cells)
Parathyroid gland	none
Peripheral nerves	none
Prostate	none
Salivary gland	cytoplasmic
Small intestine	cytoplasmic (enterochromaffin cells)
Spleen	none
Skeletal muscle	none
Skin	cytoplasmic
Stomach	cytoplasmic (glands)
Testis	none
Thyroid gland	none
Thymus gland	cytoplasmic (Hassal’s corpuscle)
Tonsil	none
Uterine cervix	none

ROR1 protein expression across a panel of protein lysates of 14 post-mortem tissues from 2 previously healthy children were analyzed by Western blotting with goat anti-human ROR1 pAbs followed by horse radish peroxidase (HRP)-conjugated donkey anti-goat IgG pAbs. A firm 120-kDa band, which we previously correlated with cell surface ROR1 in primary CLL cells [13], B-ALL cell lines (Fig. 2C), and primary B-ALL blasts (data not shown), was only detected in the positive control (Burkitt lymphoma cell line CA-46), but not in normal pediatric tissues (Fig. 5B). As noted previously for normal adult tissues [13], a 50-kDa band, which was not detectable with the secondary antibody alone (data not shown), was also prominent in the majority of normal pediatric tissues. It is possible that the cytoplasmic staining we observed in normal adult tissues by IHC (Table 2 and Fig. 5A) is due to a 50-kDa ROR1 protein consisting of the three extracellular domains and (i) encoded by ROR1 mRNA isoform 2, or (ii) post-translationally derived from the 120-kDa ROR1 protein, or (iii) a combination of both. In addition, Western blotting detected a 150-kDa band in pediatric uterus and a 75-kDa band in pediatric spleen (Fig. 5B) that we cannot exclude to be cross-reactivity of the goat anti-human ROR1 pAb.

### Cell Surface ROR1 Mediates Antibody Internalization

**Figure 6:**
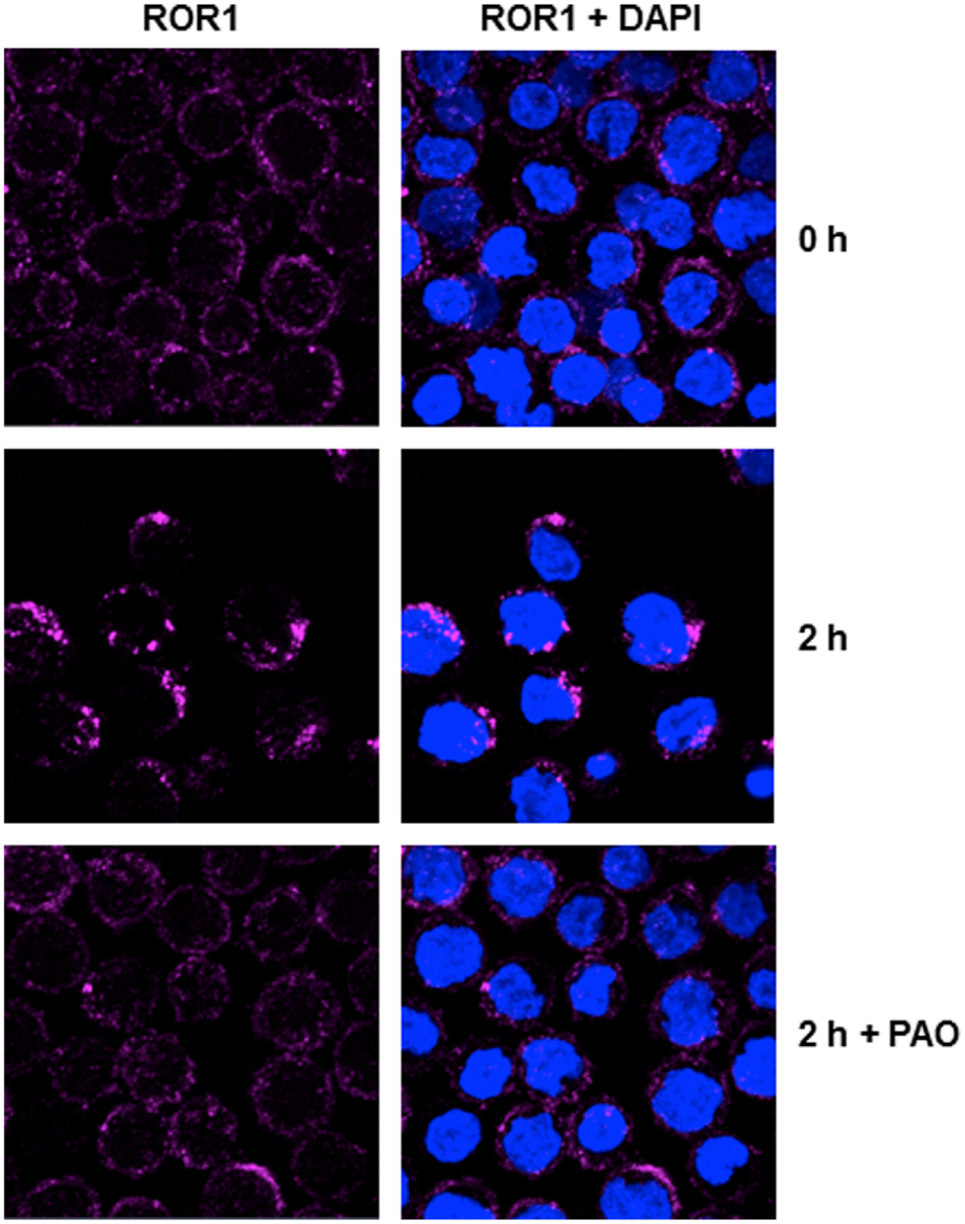
ROR1-mediated antibody internalization. Confocal immunofluorescence microscopy demonstrated internalization of mouse anti-human ROR1 mAb 2A2 by pre-B ALL cell line 697. The cells were incubated with mAb 2A2 for 1 h on ice followed by incubation at 37°C for 0–2 h in the absence or presence of PAO. Subsequently, the cells were fixed, permeabilized, stained with DyLight 649-conjugated goat anti-mouse IgG pAbs, co-stained with DAPI, and analyzed with a Zeiss LSM 510 UV confocal microscope (objective 63×). In the absence of PAO, mAb 2A2 (pink) was found uniformly on the cell surface at 0 h (top panel) and predominantly in cytoplasmic clusters at 2 h (middle panel). No internalization at 2 h was seen in the presence of PAO (bottom panel). DAPI staining the nucleus is seen in blue. (Original magnification×2).

To assess the scope of mAb-based therapies targeting cell surface ROR1 in pediatric B-ALL, we next investigated antigen-mediated internalization, a prerequisite for antibody-drug conjugates and immunotoxins. Although we previously demonstrated that cell surface ROR1 can mediate the internalization of pAbs and mAbs in primary CLL cells and mantle cell lymphoma cell lines [13], [25], [26], it was important to confirm this in the setting of pediatric ALL. Furthermore, we sought to improve on our published data that were based on indirect evidence through flow cytometry by providing direct evidence for internalization through confocal immunofluorescence microscopy. For this, we incubated pre-B-ALL cell line 697 with mAb 2A2 on ice followed by incubation for 0–2 h at 37°C in the absence or presence of endocytosis inhibitor phenylarsenine oxide (PAO). The cells were then fixed and permeabilized to allow subsequent staining with DyLight 649-conjugated goat anti-mouse IgG pAbs. As shown in Fig. 6, mAb 2A2 revealed uniform cell surface localization on B-ALL cells at 0 h (top panel) or at 2 h in the presence of PAO (bottom panel). By contrast, following incubation for 2 h at 37°C in the absence of PAO, mAb 2A2 was predominantly found in cytoplasmic clusters that resembled endosomes (middle panel), indicating antibody internalization mediated by cell surface ROR1. Although the cytoplasmic clusters resembled endosomes and were not observed for a mouse anti-human CD19 mAb, which remained on the cell surface under the same conditions, internalized ROR1 did not co-localize with Early Endosome Antigen 1 (EEA1; data not shown). This suggests that the antigen/antibody complex was internalized through EEA1-negative endosomes. Similar results were obtained for ROR1-expressing primary B-ALL blasts (data not shown).

## Discussion

To investigate the utility of ROR1 as a target for mAb therapy of pediatric B-ALL, we analyzed cell surface ROR1 expression across ALL subtypes and in normal adult and pediatric tissues. Our study is a comprehensive analysis of cell surface ROR1 expression in B-ALL, including 14 ALL cell lines and 56 primary ALL blasts with a variety of immunophenotypes and genotypes that represent the heterogeneity of pediatric ALL. Notably, ROR1 expression was found to be variable across and within subtypes except for the E2A-PBX1 genotype that demonstrated uniform ROR1 expression. Patients with t(1;19) pre-B ALL generating the oncogenic fusion protein E2A-PBX1 were once considered to have poor prognosis and high risk of central nervous system relapse, but intensification of chemotherapy has improved outcome, although with increased risks of long-term toxicities [27]. Whereas our results showing uniform ROR1 expression in all E2A-PBX1 patients at the mRNA and protein level are in line with recently published findings [18], they unveil that cell surface ROR1 expression is not limited to this B-ALL subtype. In fact, most of the E2A-PBX1-negative/ROR1+ patients revealed higher levels of cell surface ROR1 than E2A-PBX1+/ROR1+ patients. Furthermore, cell surface ROR1 expression was not only found in newly diagnosed, but also in pediatric B-ALL patients with multiply relapsed chemorefractory disease. Collectively, these findings have important implications for the recruitment of pediatric B-ALL patients to future clinical trials that investigate mAb-based therapies targeting ROR1. We propose that cell surface expression of ROR1 detected by flow cytometry should be used as inclusion criterion for all subtypes of B-ALL. Another significant finding of the current study in support of clinical trials is the virtual absence of ROR1 protein on the surface of normal adult and pediatric tissues and circulating cells. This is consistent with studies in rats and mice showing downregulation of ROR1 postnatally [10], [28], [29]. Nonetheless, cell surface ROR1 expression previously reported on a pre-B cell stage in the bone marrow [17], [18] and on adipocytes [17], the latter of which was confirmed in our study, as well as potential low level cell surface expression in certain pediatric organs (Fig. 5B) should be cautiously considered to assess and recognize possible on-target toxicities in mAb-based therapies targeting ROR1. Preclinical investigations that utilize mAbs to conserved epitopes in human, nonhuman primate, and mouse ROR1 [25] will allow to monitor any short-term and long-term toxicities associated with targeting a normal pre-B cell stage and adipocytes. Further, our study provides evidence for broad cytoplasmic expression of ROR1 (Table 2 and Fig. 5A) along with the presence of ROR1 mRNA isoform 1 (Fig. 1A) and a 50-kDa ROR1 protein (Fig. 5B) [13], which likely consists of the three extracellular domains, in normal adult and pediatric tissues. Although cytoplasmic ROR1 may not interfere with mAb-based therapies targeting cell surface ROR1, its origin and function needs to be addressed in future studies.

Aside from restricted expression, ideal targets of therapeutic utility are functionally implicated in the pathophysiology of a disease [30]. If a potential target is a “passenger” rather than a “driver”, selective pressure may lead to its downregulation without correcting disease progression. Although the finding that cell surface ROR1 is not uniformly expressed in pediatric B-ALL (in contrast to CLL) rules out an essential role across all B-ALL subtypes and although ROR1 mRNA expression is not associated with aggressive disease (similar to CLL), several lines of evidence point at a functional implication. First, previous studies have established ROR1 as a survival kinase [22], [31], [32] or pseudokinase [33] whose silencing with siRNA significantly interferes with *in vitro* and *ex vivo* survival of cell lines and primary cells, including primary B-ALL blasts from E2A-PBX1+ ROR1+ patients [20]. Second, the immunophenotypic and genotypic heterogeneity of ALL implies addiction to diverse survival signaling pathways, some of which may involve ROR1 as indicated by its uniform expression in the E2A-PBX1 subtype and our finding that >90% of primary B-ALL blasts from ROR1+ pediatric B-ALL patients express ROR1 with homogeneous cell surface density. Third, cell surface expression of ROR1 has been detected in an increasingly diverse portion of hematologic and solid malignancies [34]. This observation points to a potentially broader functional implication of ROR1 in cancer.

Accessibility of target cells and availability of effector proteins and cells in blood and bone marrow provide a suitable environment for mAb-based therapies of hematologic malignancies including pediatric B-ALL [3]. Results from a Children’s Oncology Group trial using anti-CD20 mAb rituximab and anti-CD22 mAb epratuzumab alone or in conjunction with chemotherapy in relapsed B-ALL indicate that therapeutic mAbs can be safely administered in children [4]. However, normal B cells that express both CD20 and CD22 are also depleted by these mAbs, leading to potentially prolonged immunosuppression. We envision that the absence of ROR1 on normal B cells and its virtual absence on other normal circulating cells and tissues will allow targeting of pediatric B-ALL with minimal side effects. In fact, endogenous anti-ROR1 pAbs that were detected in CLL patients after vaccination with autologous CD154-transduced CLL cells [14] as well as after treatment with lenalidomide [35] suggest that anti-ROR1 mAbs are safe. We have developed a panel of mouse and rabbit mAbs that bind ROR1 with high specificity and affinity [25], [26]. Initial studies based on this panel suggest that ROR1, at the relatively low cell surface densities found in CLL and B-ALL, may be a preferred target for armed rather than naked mAbs. Based on its ability to mediate mAb internalization, ROR1-targeting antibody-drug conjugates and immunotoxins may be of particular interest [26]. In addition, the recruitment of cytotoxic T cells through ROR1-targeting chimeric antigen receptors [17] and bispecific antibodies similar to blinatumomab [8] are promising strategies for preclinical and clinical investigations.

In conclusion, cell surface expression in pediatric B-ALL along with its virtual absence from normal tissues and circulating cells makes ROR1 a promising target for mAb-based therapies. Our study provides a rationale for further preclinical and clinical investigations of anti-ROR1 mAbs and their derivatives in the treatment of high risk and chemorefractory pediatric B-ALL in an attempt to improve relapse-free survival rates and overcome short-term and long-term toxicities associated with current treatment regimens.

## Materials and Methods

### Cells and Tissues

Pre-B ALL cell lines 380, 697, KASUMI-2, KOPN-8, MHH-CALL-3, NALM-6, RCH-ACV, RS4;11, SEM, SUP-B15, and T-ALL cell line MOLT-16 were purchased from the German Resource Center for Biological Materials (DSMZ; Braunschweig, Germany). Pre-B ALL cell line REH and Burkitt lymphoma cell line CA-46 were from the American Tissue Culture Collection (ATCC; Manassas, VA) and pre-B ALL cell line EU-1 was kindly provided by Dr. Muxiang Zhou (Emory University, Atlanta, GA) [36]. All cell lines were maintained in RPMI 1640 medium supplemented with 10% fetal bovine serum (FBS), GlutaMAX, and penicillin-streptomycin (all from Invitrogen, Carlsbad, CA). Bone marrow or peripheral blood samples of 56 pediatric patients with ALL were obtained from tissue banks at the NCI, NIH (Bethesda, MD) and Johns Hopkins Hospital (Baltimore, MD). Patient samples were obtained after written informed consent was obtained and approved by the National Cancer Institute Institutional Review Board and the Johns Hopkins University School of Medicine Institutional Review Board. The samples were cryopreserved in RPMI 1640 medium supplemented with 10% FBS and 10% dimethyl sulfoxide and were thawed immediately prior to the experiments. Frozen tissues from 14 organs of two deceased healthy pediatric female patients ages 8.9 and 4.8 years were obtained from the NICHD Brain and Tissue Bank for Developmental Disorders at the University of Maryland School of Medicine (Baltimore, MD) (http://medschool.umaryland.edu/btbank/). An unstained paraffin normal tissue array slide with 99 cores comprising 32 adult normal tissues from 47 cases (FDA999) was purchased from US Biomax (Rockville, MD).

### Gene Expression Profiling

Relative ROR1 expression was calculated as median-centered log2 expression levels from a published microarray dataset (http://pob.abcc.ncifcrf.gov/cgi-bin/JK) based on probe 205805_s_at (ROR1 isoform 1) on the GeneChip Human Genome U133A Array (Affymetrix, Santa Clara, CA). The dataset includes diagnostic samples of 132 newly diagnosed ALL patients treated at St. Jude Children’s Research Hospital (http://www.stjuderesearch.org/data/ALL1)[21]. The patients were grouped according to the commonly recurring chromosomal translocations, namely t(1;19) that generates the fusion protein E2A-PBX1, t(11;23) with MLL rearrangement, t(9;22) with BCR-ABL fusion protein, t(12;21) with TEL-AML1 fusion protein, hyperdiploidy, T-ALL, and all others as complex. Normal tissues represented various post-mortem samples from different age groups (2.3–40.9 years). Where indicated, results were tested for statistical significance using Prism 5.01 (GraphPad Software, La Jolla, CA). Mann-Whitney test and Kruskal-Wallis one-way ANOVA test were used for non-parametric variables. Results were considered significant for p<.05.

### Flow Cytometry

Cell surface expression of ROR1 was analyzed using multiparameter flow cytometry on a FACSCalibur instrument (BD Biosciences Immunocytometry Systems, San Jose, CA). Cell lines and primary cells were washed twice with phosphate-buffered saline (PBS) containing 2% FBS (FACS buffer) and resuspended at a concentration of 1×10^7^/ml. Primary cells were blocked with 100 µg/ml purified polyclonal human IgG (Invitrogen) for 15 min at room temperature and washed with FACS buffer. Approximately 5×10^5^ cells were incubated with 1 µg/ml of affinity purified mouse anti-human ROR1 IgG1 mAb 2A2 [26] or, as isotype control, ChromPure Mouse IgG (Jackson Immunoresearch Laboratories, West Grove, PA) for 1 h on ice. After two washes, the cells were incubated with DyLight 649-conjugated goat anti-mouse IgG affinity purified polyclonal antibodies (pAbs) (Jackson Immunoresearch Laboratories) at a dilution of 3 µg/ml for 1 h on ice. After two washes, propidium iodide was added to exclude dead cells from analysis. Primary cells were also stained with anti-CD19-FITC and anti-CD10-PE mAbs (BD Biosciences) to gate CD19+ CD10+ B-ALL blasts, which were >90% in all patients samples, except for MLL cases, where a CD19+ CD10-negative gate was used. Anti-CD3-FITC and anti-CD5-PE mAbs (BD Biosciences) were used to gate T-ALL blasts. A total of 20,000 gated events per sample were collected using CellQuest software (BD Biosciences) and data were analyzed using FlowJo analytical software (Tree Star, Ashland, OR). To determine the cell surface density of ROR1, mAb 2A2 was conjugated to phycoerythrin (PE) using the PhycoLink Activated R-Phycoerythrin conjugation kit according to the manufacturer’s instructions (ProZyme, Hayward, CA). Cells were incubated with mAb 2A2-PE for 1 h on ice and then washed three times with FACS buffer. The QuantiBRITE PE kit (BD Biosciences) was used as a standard to calibrate the number of ROR1 molecules per cell. QuantiBRITE PE beads were acquired on a FACSCalibur instrument on the same day and with the same instrument settings used for the cells, generating a standard curve with the Quantitative Calibration option in CellQuest software. Using the slope and intercept of the standard curve, the antibody binding capacity of the cells was determined according to the manufacturer’s instructions.

### Western Blotting

Total cell lysates from each cell line, patient samples and normal tissues were prepared using lysis buffer [10 mM Tris-HCl (pH 8.0), 130 mM NaCl, 1% (v/v) Triton X-100, 5 mM EDTA, and protease inhibitor cocktail (Thermo Scientific Pierce, Rockford, IL)] as previously described [13]. After five washes, the cell pellets were resuspended in lysis buffer at 1×10^7^ cells/ml and incubated for 1 h on ice followed by centrifugation at 20,000 g and 4°C for 30 min, and the protein concentration in the supernatant was determined using the BCA Protein Assay kit (Thermo Scientific Pierce). Cell lysates (5–10 µg per lane) or normal tissue lysate (20 µg per lane) were separated on a NuPAGE Novex 4%–12% Bis-Tris Gel (Invitrogen) under reducing condition. Fc-ROR1 protein (250 ng) [25] and cell lysate from CD19+ normal B cells were used as positive and negative controls, respectively. After SDS-PAGE, the proteins were transferred to a Hybond ECL nitrocellulose membrane (GE Healthcare, Piscataway, NJ). The membranes were incubated overnight at 4°C with 1× Western Blocking Buffer (Roche Applied Science, Indianapolis, IN) in Tris-buffered saline (TBS) and then incubated for 1 h at room temperature with 200 ng/ml goat anti-human ROR1 pAbs (R&D Systems, Minneapolis, MN). The membranes were washed three times with 0.5% (v/v) Tween 20 (Sigma-Aldrich, St. Louis, MO) in TBS followed by incubation with a 1∶10,000 dilution of HRP-conjugated donkey anti-goat IgG pAbs (Jackson Immunoresearch Laboratories) for 1 h at room temperature. After three additional washes with 0.5% (v/v) Tween 20 in TBS, the proteins were detected by chemiluminescence using SuperSignal West Pico Substrate (Thermo Scientific Pierce). To confirm uniform loading of protein, the membranes were then stripped with Western blot stripping buffer (Thermo Scientific Pierce) and re-probed with 200 ng/ml of rabbit anti-human glyceraldehyde 3-phosphate dehydrogenase (GAPDH) pAbs (Imgenex, San Diego, CA) followed by HRP-conjugated goat anti-rabbit IgG pAbs (Imgenex).

### Immunohistochemistry

FFPE slides prepared from cell lines and tissue microarray were stained on a Bond Autostainer (Leica Microsystems, Wetzlar, Germany). Using heat-induced epitope retrieval with citrate buffer, the slides were blocked with 5% (v/v) normal rabbit serum in PBS and incubated with goat anti-human ROR1 pAbs or normal goat IgG (R&D Systems) at 0.9 µg/ml for 30 min (cell lines) or 2.25 µg/ml for 1 h (tissue microarray). Detection was accomplished using Novocastra biotinylated rabbit anti-goat IgG pAbs and the Novocastra Bond Intense R Detection kit (Leica Microsystems).

### Confocal Immunofluorescence Microscopy

Approximately 1×10^6^ cells were first blocked with 100 µg/ml purified polyclonal human IgG for 15 min at room temperature and then incubated for 1 h on ice with 5 µg/ml mAb 2A2. The cells were washed three times with FACS buffer to remove unbound antibody and then either placed on ice or incubated at 37°C for 30 min, 60 min, 90 min, or 2 h to facilitate internalization. A duplicate sample for the 2-h time point was incubated with or without 10 µM PAO (Sigma-Aldrich) [12]. The cells were washed and fixed with 3% (w/v) paraformaldehyde (Electron Microscopy Sciences, Hatfield, PA) for 15 min at room temperature and then incubated in permeabilization buffer containing 0.05% (w/v) saponin, 1 M glycine, 0.02% (w/v) sodium azide (all from Sigma-Aldrich) and 5% (v/v) FBS for 15 minutes at room temperature. All subsequent steps were done using the permeabilization buffer. The cells were then incubated for 1 h with DyLight 649-conjugated goat anti-mouse IgG affinity purified pAbs at a concentration of 6 µg/ml, costained with 4′,6-diamidino-2-phenylindole (DAPI; Vector Laboratories, Burlingame, CA), and washed. Pictures were acquired using a Zeiss LSM 51UV confocal microscope using a 63× oil immersion lens and Zeiss LSM Image Browser software.
